# Evaluation of Patients With Iron Deficiency Anaemia Referred via the Colorectal Cancer Two-Week Pathway in a District General Hospital: A Retrospective Cohort Study

**DOI:** 10.7759/cureus.85502

**Published:** 2025-06-07

**Authors:** Ali Javaid, Kehkashan Anwar, Shariq Sabri, Amir Saleem

**Affiliations:** 1 General Surgery, Tameside and Glossop Integrated Care National Health Service (NHS) Foundation Trust, Manchester, GBR; 2 General Surgery, Tameside General Hospital, Manchester, GBR; 3 Gastroenterology, Tameside and Glossop Integrated Care National Health Service (NHS) Foundation Trust, Manchester, GBR

**Keywords:** 2-week-wait referral, colorectal cancer, gi cancer, iron-deficiency, iron deficiency anaemia

## Abstract

Background

Iron deficiency anaemia (IDA) is a frequent clinical presentation in both primary and secondary care and is a recognised red-flag symptom for underlying gastrointestinal malignancy, particularly colorectal cancer. Prompt and efficient investigation is essential to identify significant pathology while minimising unnecessary procedures.

Objectives

To evaluate the clinical outcomes of patients referred under the two-week wait (2WW) pathway for suspected colorectal cancer due to IDA in a district general hospital setting.

Methods

A retrospective review was conducted of 50 consecutive patients referred with IDA via the 2WW pathway. Data collected included demographics, triage method, investigations undertaken, procedures performed, and final diagnoses.

Results

A total of 50 patients were included. The mean age was 63.2 years (SD ±10.7), and 60% (n=30) of patients were female, while males (40%, n=20) constituted the rest of the cohort. All patients underwent initial triage by a specialist nurse, with 42% (n=21) referred directly for endoscopy, while the remainder (n=29, 58%) were seen in outpatient clinics. Bidirectional endoscopy was performed in 82%-94% of cases, with additional CT imaging in selected patients. Significant findings included one case (n=1, 2%) of colorectal cancer, 12% (n=6) diagnosed with inflammatory bowel disease or coeliac disease, and 8% (n=4) entered polyp surveillance. Most patients (n=35, 70%) were discharged after reassurance.

Conclusions

Structured triage led by specialist nurses enables efficient use of endoscopy and outpatient resources. While the diagnostic yield for malignancy was low (n=1, 2%), a notable proportion of patients had alternative significant findings. These results support a comprehensive diagnostic approach for IDA but highlight the need for improved risk stratification tools to optimise resource utilisation and patient outcomes.

## Introduction

Anaemia is a worldwide issue affecting approximately one-third of the world’s population, and iron deficiency is the predominant cause. IDA is characterised by haemoglobin levels below the lower limit as defined by performing labs for that specific age group along with low iron levels. IDA affects 2-5% of adults globally, with recent estimates indicating that anaemia affects almost 1.62 billion individuals worldwide [1], with gastrointestinal (GI) blood loss accounting for 30-50% of cases in men and postmenopausal women [2]. The main symptoms associated with anaemia include chronic fatigue, impaired cognitive function, and diminished well-being. Adequate management of IDA can significantly improve general well-being and quality of life.

IDA can result from inadequate intake, reduced absorption, increased demand, and loss of the body [3]. IDA is also a key indicator for colorectal cancer, requiring urgent referral via the UK’s 2WW pathway [4]. National guidelines, including those from the British Society of Gastroenterology (BSG) and the National Institute for Health and Care Excellence (NICE), advocate for bidirectional endoscopy in patients with unexplained IDA, particularly in older men and postmenopausal women [4,5].

In clinical practice, however, the approach to triage and diagnostic workup varies across institutions. The use of non-invasive markers such as ferritin and faecal immunochemical testing (FIT) has increasingly been adopted to support clinical decision-making.

Objective

This study aims to evaluate the management of IDA patients referred via the 2WW pathway at a District General Hospital (DGH), assessing compliance with guidelines and diagnostic outcomes.

## Materials and methods

Study design and setting

A retrospective observational study was conducted at Tameside and Glossop NHS Trust, reviewing the medical records of 50 consecutive adult patients referred under the 2WW colorectal cancer pathway for the evaluation of iron deficiency anaemia (IDA). The study period spanned from March 2024 to April 2024.

Inclusion criteria

All patients 18 years and over referred on the 2WW colorectal pathway specifically for unexplained iron deficiency anaemia (defined as Hb <130 g/L in men and <120 g/L in women, with/or ferritin <30 µg/L) were included.

Exclusion criteria

All IDA referrals with known GI malignancy or those not referred on the 2 WW pathway were excluded.

Data collection

Data were extracted from three main sources: paper clinical notes, the electronic patient record system (Lorenzo), and the Unisoft GI reporting tool. The data collected included demographics (age, sex), haemoglobin (Hb) and ferritin levels, FIT results, triage outcomes, types of diagnostic investigations undertaken, and final clinical outcomes.

Statistical analysis

Data were entered into Microsoft Excel (Redmond, USA) and analysed using IBM Corp. Released 2022. IBM SPSS Statistics for Windows, Version 28. Armonk, NY: IBM Corp. Descriptive statistics were used to summarise demographic and clinical characteristics. Categorical variables were expressed as frequencies and percentages. Continuous variables were presented as mean ± standard deviation or median with interquartile range (IQR), depending on data distribution. Group comparisons were made using Chi-square or Fisher’s exact test for categorical variables and t-tests or Mann-Whitney U tests for continuous variables. A p-value of <0.05 was considered statistically significant.

Ethical considerations

The study was conducted in accordance with institutional ethical standards and aligned with the Declaration of Helsinki, as it was a retrospective review of anonymised patient data, formal ethical approval was deemed not required.

## Results

Patient demographics and triage outcomes

**Figure 1:**
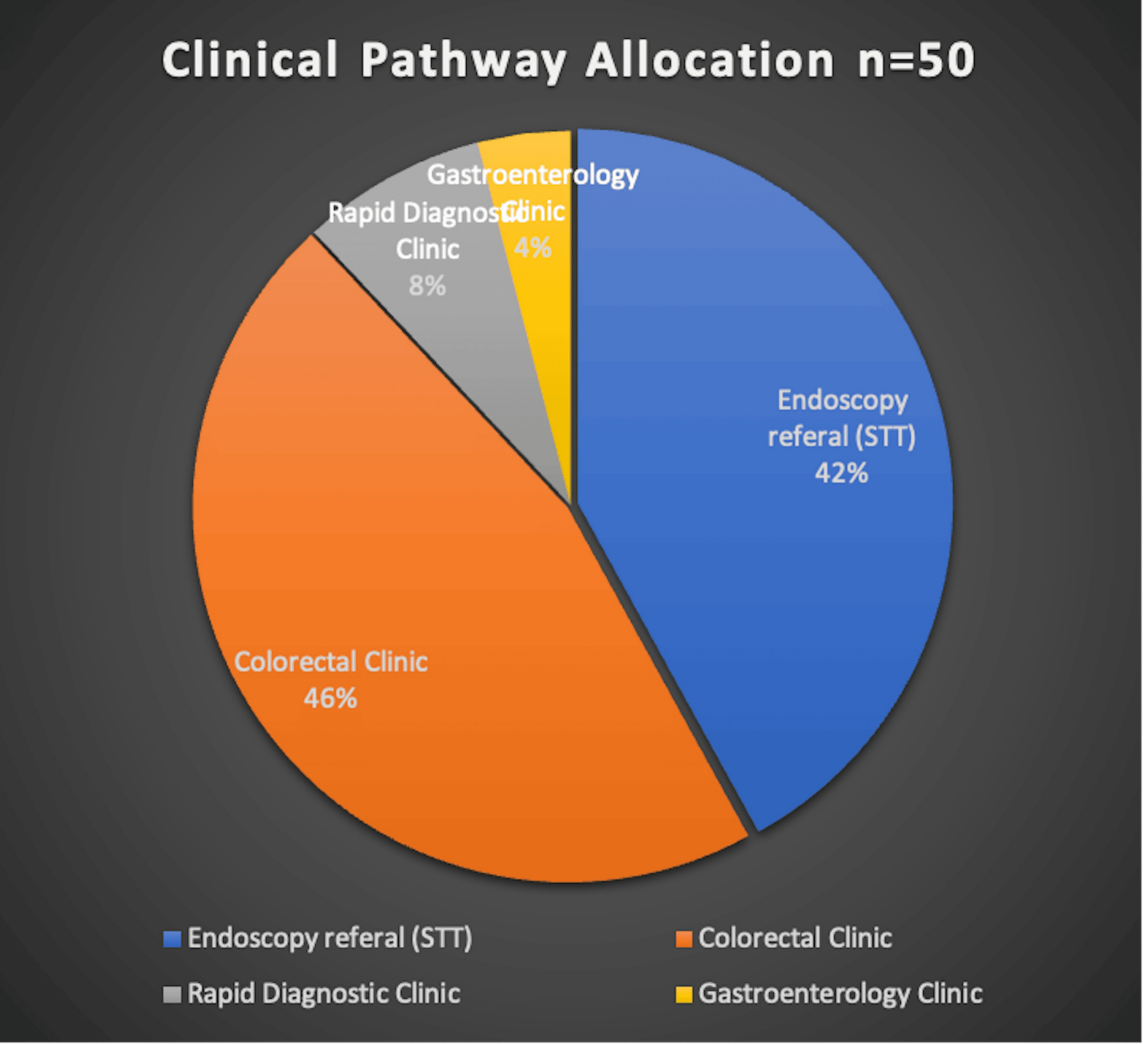
Clinic pathway allocation (N = 50) The image is created by the author. Distribution of patients across referral pathways shown as a percentage of the total cohort with respective N values. Data are expressed as N (%).

A total of 50 patients on the two-week pathway with iron deficiency anaemia (IDA) were reviewed, comprising 20 males (40%) and 30 females (60%). Although a higher proportion of females was observed, the difference was not statistically significant (χ² = 2.00, p = 0.157). The age range was 24-93 years (mean 63.22; mode 80). All patients were triaged by a specialist nurse, with 21 (42%) referred directly for endoscopic evaluation. The remaining 29 (58%) were seen in clinics. This distribution lacked statistical significance (χ² = 1.28, p = 0.258). Out of 29 (58%) seen in the clinic, 23 (46%) were assessed in the colorectal clinic, 4 (8%) in the Rapid Diagnostic Centre (RDC), and 2 (4%) in the gastroenterology clinic (Figure 1).

Baseline investigations available at triage

Basic investigations available at triage included haemoglobin (Hb) levels, checked in all 50 patients (range 5.1-13.8 g/dL, mean = 9.8, SD ± 1.6 g/dL), ferritin measurements in 46 patients (92%; 25/46, 54.3% low), and faecal immunochemical testing (FIT) in 47 patients (94%; 21/47, 44.7% normal).

Diagnostic investigations performed

Colonoscopy was performed on 41 patients (82%), 2 (4.9%) of whom had undergone the procedure within the preceding two years. Gastroscopy was completed in 47 patients (94%), with 11 (23.4%) having had a prior gastroscopy in the last 2 years. Seven patients (14%) underwent CT colonography or CT scans due to comorbidities or patient preferences, while 14 (28%) had additional CT scans alongside endoscopy for symptoms such as weight loss or abdominal pain (Table 1).

**Table 1:** Distribution of clinic pathways and diagnostic outcomes Data are represented as N (%). Chi-square tests were used for categorical variables. A p-value of < 0.05 was considered statistically significant.

Category	N (%)	Statistical Test	Test Statistic	p-value
Gender (Male vs. Female)	20 (40%) vs. 30 (60%)	Chi-square test	χ² = 2.00	0.157
Direct endoscopy vs. Clinic visit	21 (42%) vs. 29 (58%)	Chi-square test	χ² = 1.28	0.258
Colonoscopy performed	41/50 (82%)	–	–	–
Gastroscopy performed	47/50 (94%)	–	–	–
CT/CTC performed	7/50 (14%)	–	–	–
Additional CT imaging	14/50 (28%)	–	–	–
Diagnosed malignancy	1/50 (2%)	–	–	–

Clinical outcomes

**Figure 2:**
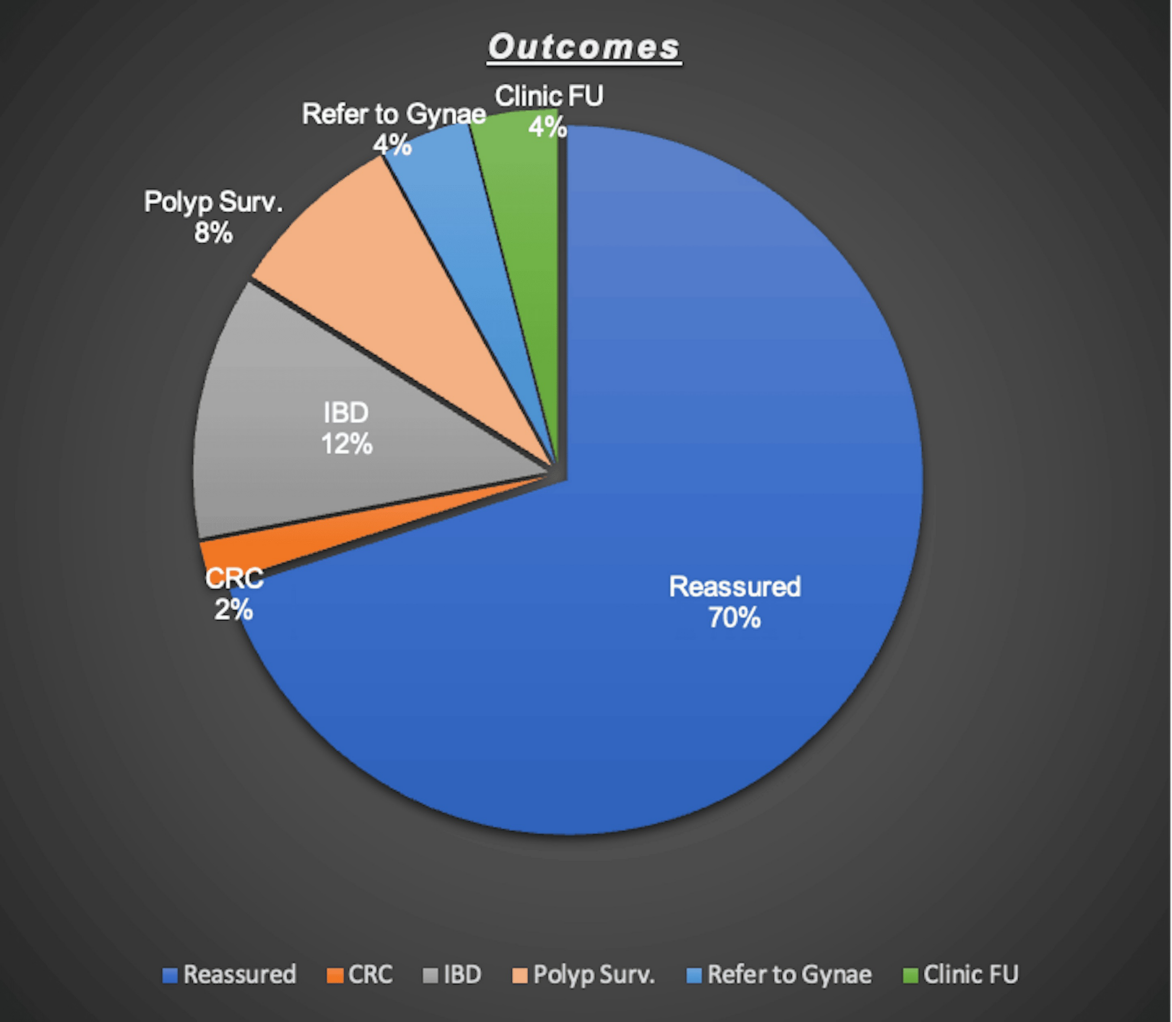
Final patient outcomes (N = 50) The image is created by the author. Outcomes post-diagnostic evaluation are represented in N (%). Data are categorical and descriptive.

Most patients (35/50, 70%) were reassured and discharged. One patient (2%) was diagnosed with right-sided colonic malignancy and underwent surgical resection. Six patients (12%) were followed by gastroenterology for inflammatory bowel disease (IBD) or coeliac disease; 4 (8%) entered a polyp surveillance pathway; 2 (4%) were referred to gynaecologists for suspected ovarian pathology; and 2 (4%) were directed to the pelvic floor clinic for symptom management (Figure 2).

Statistical analysis and significant findings

A gender-based comparison revealed a statistically significant predominance of females in the straight-to-test (STT) referral pathway compared to those assessed in the clinic before investigation (STT: 66.6% female vs. non-STT: 55.2% female; p = 0.048). This suggests that females were more likely to be referred directly for endoscopic evaluation without prior clinical consultation.

Analysis of FIT outcomes demonstrated a strong association with discharge status. Among patients with normal FIT results, the majority (n = 19) were discharged without the need for further investigations, while only a small number (n = 2) required additional follow-up (p = 0.001). This supports the utility of normal FIT results in safely triaging patients out of further invasive testing.

There was no statistically significant difference in the mean age between patients in the STT group (61.2 ± 14.5 years) and those assessed in the clinic (64.8 ± 13.1 years; p = 0.32), indicating comparable age distribution across triage pathways.

Additionally, the rate of colonoscopy completion did not differ significantly by gender (F: 25 vs. M: 16; p = 0.55), suggesting equitable access to endoscopic evaluation regardless of sex (Table 2).

**Table 2:** Comparative results table This table presents the comparative statistical analysis of key variables among patients referred on the two-week pathway with iron deficiency anaemia (IDA). Categorical variables were compared using the Chi-square test or Fisher’s exact test, while continuous variables were analysed using independent t-tests. P-values less than 0.05 were considered statistically significant.

Comparison	Group Values	Test Used	p-value	Interpretation
STT Referral by Gender	STT: 14 F (66.6%), 7 M (33.3%) Non-STT: 16 F, 13 M	Chi-square	0.048	Significant — STT referrals higher in females
FIT Result vs. Follow-up Outcome	Normal FIT: Discharged = 19 Further actions = 2	Chi-square	0.001	Strong association — normal FIT → discharge
Age in STT vs. Clinic group	STT: 61.2 ± 14.5 yrs Clinic: 64.8 ± 13.1 yrs	Independent t-test	0.32	Not significant — similar mean age
Colonoscopy by Gender	Colonoscopy completed: F = 25, M = 16	Chi-square	0.55	Not significant — no gender difference

## Discussion

IDA is a common finding in clinical practice, especially in older adults, and is frequently associated with GI blood loss, prompting investigation for possible underlying malignancy or other GI pathology. The 2WW pathway aims to expedite diagnostic workup for suspected colorectal cancer, including patients with IDA, due to the recognised association between unexplained anaemia and GI malignancies [4-6]. Our evaluation of 50 patients referred via the 2WW pathway in a district general hospital provides insights into the effectiveness of triage, diagnostic yield, and patient outcomes while reflecting on the broader implications of managing IDA in secondary care.

In this cohort, all patients were triaged by a specialist nurse, with 42% (n=21) directly referred for endoscopic procedures and the remainder undergoing clinical assessment in various speciality clinics. This model aligns with contemporary guidance advocating early endoscopic evaluation in high-risk patients, particularly men and postmenopausal women with confirmed IDA [2]. The triage system appeared to function effectively in prioritising patients appropriately, as evidenced by the majority undergoing complete endoscopic workup (82%, n=41 colonoscopy, 94%, n=47 gastroscopy). The presence of a specialist nurse-led triage system is a practical solution in the face of workforce pressures and allows timely clinical decisions based on initial investigations such as haemoglobin, ferritin, and FIT results [4].

Ferritin levels and FIT were measured in 92% (n=46) and 94% (n=47) of patients, respectively. Low ferritin was present in 54.3% (n=25) of those tested, reinforcing its value in confirming iron deficiency as the aetiology of anaemia. FIT, now widely adopted in primary and secondary care for colorectal cancer (CRC) risk stratification, was normal in 44.7% (n=21) of tested patients. While FIT has demonstrated a high negative predictive value (NPV) for CRC [7], it is not always a correct indicator of disease. False negatives can occur in cases of right-sided or non-bleeding tumours and patients with intermittent bleeding. The single cancer diagnosis in our cohort (2%, n=1) reinforces FIT's utility in combination with clinical judgement rather than as a standalone test.

The comprehensive use of colonoscopy and gastroscopy in this cohort reflects adherence to recommended best practices. The BSG and NICE recommend bidirectional endoscopy in patients with unexplained IDA to evaluate both upper and lower GI tracts [4,5]. In our cohort, 11 patients had previous gastroscopies within the preceding two years, prompting a review of the necessity for repeat upper GI endoscopy in the absence of new symptoms. This is an area that requires further guidance, particularly for resource optimisation and patient burden.

Fourteen per cent of patients underwent CT colonography or cross-sectional imaging in place of colonoscopy due to comorbidities or personal preferences. This shows clinical pragmatism, as colonoscopy may be contraindicated or refused by frail or elderly patients. CT colonography offers reasonable sensitivity for CRC detection and is considered a suitable alternative in such scenarios [8]. Additionally, 28% (n=14) underwent adjunctive CT scans due to alarm features such as weight loss or abdominal pain, highlighting the importance of holistic assessment beyond anaemia alone.

The majority of patients (70%, n=35) were reassured and discharged following a normal diagnostic workup, consistent with results from different studies showing low malignancy yield in patients with IDA, especially in females and younger patients [9]. However, one patient (2%, n=1) was diagnosed with right-sided colonic malignancy. Although this is a relatively low figure, it justifies the aggressive investigative strategy given the high stakes of delayed cancer diagnosis. Other significant findings included inflammatory bowel disease (IBD), coeliac disease, and colonic polyps, which accounted for 12-20% of referrals. These results support the view that while most patients with IDA may not have cancer, a considerable proportion will have clinically relevant pathology warranting follow-up.

Our findings are broadly consistent with published studies. For example, different UK studies found low CRC detection rates in IDA patients referred via the 2WW pathway, with significant non-malignant pathology (e.g., IBD, angiodysplasia) observed [9,10]. Our lower cancer detection rate may reflect a smaller sample size.

Our findings support the continued use of triage-led bidirectional endoscopy in IDA patients referred on the 2WW pathway, especially in those at higher risk (e.g., older males, postmenopausal women, low ferritin, positive FIT). However, a significant proportion of patients had normal investigations, raising the question of whether enhanced risk stratification tools could reduce unnecessary procedures while maintaining diagnostic accuracy.

Study limitations

The limitations of this study include the small sample size, its retrospective design, and the absence of a validated risk assessment tool, all of which may introduce selection and documentation bias. Furthermore, reliance on previously recorded clinical data may have led to underreporting of relevant clinical or diagnostic information.

## Conclusions

This evaluation of patients referred on the 2WW pathway for IDA at a district general hospital highlights the need for an effective and structured triage mechanism which can provide comprehensive, timely investigations in managing this common yet challenging condition. The use of ferritin and FIT at the point of referral supported risk stratification and prioritisation, while the high rate of endoscopic investigations ensured thorough evaluation of potential gastrointestinal sources of anaemia. Although the cancer detection rate was low (2%, n=1), significant non-malignant pathology, such as inflammatory bowel disease and coeliac disease, was found in 20% of patients, highlighting the value of the diagnostic process. The data also demonstrate the need for refined referral criteria or risk-scoring models to reduce unnecessary procedures while maintaining diagnostic accuracy.

Overall, this analysis supports a multidisciplinary, patient-centred approach and highlights the need for ongoing refinement in the management of IDA within cancer referral pathways.
